# Kinky Sex and Deliberate Partner Negotiations: Case Studies of Canadian Transgender Men Who Have Sex with Men, Their HIV Risks, Safer Sex Practices, and Prevention Needs

**DOI:** 10.3390/ijerph191811382

**Published:** 2022-09-09

**Authors:** Renato M. Liboro, Charles Fehr, George Da Silva

**Affiliations:** 1 Department of Psychology, University of Nevada, Las Vegas, NV 89154, USA; 2 Centre for Addiction and Mental Health, Toronto, ON M5S 2S1, Canada

**Keywords:** transgender men who have sex with men, resilience, HIV/AIDS, kinky sex, deliberate partner negotiations

## Abstract

Growing research in the last two decades has begun to investigate the HIV risks and sexual health practices of transgender men, especially as a subpopulation of men who have sex with men (MSM) that likely shares certain HIV risks and sexual health practices with cisgender MSM, the sociodemographic group that continues to be at highest risk for HIV in many developed countries since the start of the epidemic. As part of our Community-Based Participatory Research project and larger strengths-based qualitative study that was dedicated to examine multiple factors that promote resilience to HIV utilizing the perspectives and lived experiences of middle-aged and older MSM, the case studies we present in this article feature the distinct insights and experiences of three HIV-negative transgender MSM from Downtown Toronto, Ontario, Canada, who participated in our one-on-one interviews. The three case studies provide not only an enlightening snapshot of some of the specific contexts, HIV risks, safer sex practices, and HIV prevention needs of transgender MSM, but also a unique opportunity to critically reflect on the potential implications of the insights and experiences that were shared by our participants, particularly for adapting and developing current and future HIV services and programs to maximally benefit transgender MSM.

## 1. Introduction

Transgender men, or trans men, have increasingly been situated and ingrained in communities and networks of gay, bisexual, and other men who have sex with men (MSM) [1,2,3,4], the communities and networks that in recent years have continued to consistently represent the highest proportion of all reported HIV diagnoses in Canada and the US since the start of the HIV epidemic in the early 1980s [5,6]. In the last two decades, international research has recognized that many trans men strongly identify with MSM communities and networks, and, often, have sexual encounters or activities with cisgender, or cis (i.e., non-trans), MSM (CMSM) [1,4,7,8,9,10,11,12,13,14,15,16,17]. Studies have documented that as many as two-thirds of trans men identify as gay, bisexual, queer, or MSM [15,18,19], and that many trans MSM (TMSM) have been reported having CMSM as sexual partners [1,2,15,16,20,21,22,23,24,25]. The studies have also documented that trans men who have joined MSM communities may be particularly sensitive to contextual norms around HIV infection [26]. For example, in MSM communities and contexts where an HIV-positive status is more likely to be perceived as normative [9,27,28,29], trans men may believe that seroconversion will likely increase their sense of belonging [4], and may even feel the need to conceal their HIV-negative status to avoid rejection from prospective HIV-positive sexual partners [26,30]. While in most of the MSM communities and contexts where an HIV-positive status is stigmatized [28,31,32], trans men may likely be more cautious with their sexual practices to avoid acquiring an HIV infection [26].

Globally, there has been a growing interest in HIV infection disease burden and risk among transgender individuals. However, it has become apparent that the majority of prior research that has been conducted has focused on transgender women, or trans women [11,26,33,34]. This is because the risk for HIV among trans men has largely been overshadowed by the HIV risk and prevalence among trans women [4], which has led to the early finding that the research related to HIV risk and behaviors among transgender people has been almost exclusively focused on trans women since the turn of the century [16]. Despite preliminary evidence that many trans men are at a high risk for HIV, a paucity of studies on trans men’s sexual health and their HIV risks and prevention needs had been noted by many researchers [12,15,16,25,26,33], and has likely served as an impetus for conducting further research on the specific contexts, perspectives, and lived experiences of TMSM.

The prevalence rates of HIV infection and sexual risk behaviors among trans men have not been well understood because trans men have often been assumed to be primarily having sexual relations with cis women [9,12,16,23]. Due in part to this assumption, trans men have often been considered by healthcare professionals and researchers to be at low risk for HIV infection, especially in contrast to trans women [12,15]. The studies that have documented the HIV prevalence rates among trans men either have not indicated the gender of their participants’ sexual partners, or have predominantly included trans men who have identified as heterosexual [16]. The few studies that have investigated the prevalence of HIV infections among trans men participants have reported rates between 0 to 3% [15,16,17,24,35,36,37]. Since only one documented small study has included confirmed HIV test results [24], and most other studies have been based on small convenience samples [1,15], definitive conclusions regarding the prevalence of HIV infections among trans men have not been drawn from these studies’ data.

More recently, TMSM have been labeled as a key population at disproportionate risk for sexually transmitted infections, particularly Hepatitis C and HIV [1,12,38,39]. While the behavioral risk factors of TMSM have pointed to the high potential for HIV risk, recent research has indicated that the HIV prevalence among trans men has remained low [16,19,35,40], and consequently, there may still be time to intervene [24].

There have been important studies that have examined the HIV sexual risks, risk behaviors, and vulnerabilities of TMSM [1,4,16,20,41]. By 2010, the completed research with TMSM had largely been qualitative, focused on those who have been sexually active with CMSM, or descriptive of small convenience samples [15]. The qualitative studies have revealed that some TMSM engage in risk behaviors as they explore a new sexual identity, or integrate into a new community or sexual subculture, during or after gender transition. Some trans men have described a phase of post-transition shift in sexual attraction or post-transition sexual experimentation [15,41], which may involve intentional or incidental risk-taking behaviors [26,42].

Other research has shown that TMSM seem to share certain HIV-acquisition risk factors with their cis counterparts [1]. TMSM may be at risk for HIV infection when they have CMSM partners, or when they share needles for hormone or recreational drug injections [11]. TMSM have reported a variety of sexual risk behaviors, such as engaging in receptive anal and/or frontal genital sex with CMSM and inconsistent condom use [1,4,8,9,16,24,26,43]; compulsive sexual behaviors [35]; sex with anonymous or multiple partners [9,23,35]; sex with partners who were HIV-positive or of an unknown HIV status [4,9,35,37]; sex under the influence of alcohol or drugs [9,12,21,44]; and sex work [9,15,16,21,23,35]. Certain risk factors that have been identified to have a greater impact on TMSM compared to their cis counterparts include barriers to sexual negotiations with CMSM, such as unequal power dynamics; low self-esteem; and the need for gender identity affirmation [4,8,12,16,23]. TMSM have subsequently been included within the behavioral population of MSM, a well-established high risk population that has been disproportionately affected by HIV for the last four decades [23]. Additionally, research on buy-in of the use of pre-exposure prophylaxis (PrEP) among TMSM has shown that PrEP uptake among trans men has been limited, given the documented prevalent HIV risk behaviors among TMSM [11,22].

Despite the increasing number of studies involving TMSM and their sexual health in the last two decades, there are more research questions that prospectively still need to be explored [26]. Just as importantly, it is relevant to point out that many of the studies that have been conducted with TMSM since the turn of the century have focused primarily on their HIV risks and vulnerabilities [8,16,21,22,38], and very few have focused on their strengths, particularly their protective factors, strategies, and the sexual health practices that promote their resilience to HIV [16]. Broadly, there have been a growing number of studies in the past 20 years that have examined the resilience of transgender people [45,46,47,48,49,50,51,52,53,54], but none (as far as we could determine) have specifically examined the resilience of TMSM to HIV.

In this article, we present three case studies that highlight the findings of a Community-Based Participatory Research (CBPR) project and a larger qualitative study that we conducted to identify, determine, and examine the factors that promote the resilience of MSM to HIV, with a distinctive focus on the insights and lived experiences of TMSM. For the purposes of our CBPR project and case studies, our research team and community partners collaboratively established and focused the operational definition of resilience to HIV as the capacity of MSM to navigate, mitigate, avoid, address, and/or overcome the risks and adverse impacts of HIV in their lives. The three case studies we feature in this article provide not only an enlightening snapshot of some of the specific contexts, HIV risks, safer sex practices, and HIV prevention needs of transgender MSM, but also a unique opportunity to critically reflect on potential implications of the insights and lived experiences that were shared by our participants, particularly for adapting and developing current and future HIV services and programs to maximally benefit transgender MSM.

## 2. Materials and Methods

As part of a CBPR project and larger strengths-based qualitative study dedicated to exploring and investigating a variety of factors that promote resilience to HIV, based on the perspectives and lived experiences of racially and ethnically diverse, HIV-positive and HIV-negative, middle-aged and older MSM from Central and Southwestern Ontario, Canada, the case studies that we feature in this article were derived from a Big Data set that predominantly involved participants who identified as CMSM. The case studies we present in this article are focused on highlighting the insights and personal experiences of three HIV-negative TMSM who were all 40 years of age or older and were residing in Downtown Toronto, Ontario, Canada, at the time of their interviews.

In line with the guiding principles and tenets of CBPR [55], our larger qualitative study was conducted in close collaboration with our primary community partner, Realize, a community-based organization located in Toronto that addresses and responds to the diverse needs of older people at risk of or living with HIV. In partnership with Realize, our research team established a Community Advisory Board (CAB) to help determine the main aim, procedures, and conduct of our larger study. Our CAB was comprised of middle-aged and older MSM, as well as service providers from different regional not-for-profit lesbian, gay, bisexual, transgender, queer, intersex, and asexual (LGBTQIA+) agencies and AIDS service organizations (ASOs). The main aim, procedures, and conduct of our larger study were reviewed and approved by the Research Ethics Board (REB) of the Centre for Addiction and Mental Health (protocol reference number 032/2018), which is also located in Toronto. It is critical to note that middle-aged and older MSM were significantly involved in our community-engaged study, not only as the community-based agency service providers who assisted us considerably with our participant recruitment; members of our CAB; and study participants, but also as peer researchers (i.e., compensated members of our research team from the community whose identities, input, and lived/work experiences were pertinent and vital to achieving the research aim and agenda of our CBPR project, larger study, and the three case studies we feature in this article).

### 2.1. The Case Study Approach

Our choice to present and discuss the findings of the larger qualitative study of our CBPR project that was specific to TMSM as three illustrative case studies was a logical and judicious decision. A case study is a research approach that is utilized to produce an in depth, multi-faceted understanding of a complex issue in its real-life context [56]. As an established research design that is extensively used in a wide variety of disciplines, case studies can be described in various ways, the central tenet being the need to explore a phenomenon in depth and in its natural context. Each of the three case studies we describe in this article will provide an in depth look into some of the specific contexts, HIV risks, safer sex practices, and HIV prevention needs of TMSM, as well as an enlightening depiction of the insights and real-world experiences of our interview participants. Since the data in the case studies we present in this article were derived from only three participants, we exercised extra caution to help ensure our participants’ confidentiality and privacy, particularly by expressly using pseudonyms and withholding very specific descriptions of the participants and their personal contexts to keep them anonymous, as recommended by the proponents of the case study approach [56,57].

### 2.2. Participants and Procedures

The participant recruitment plan we developed and implemented was based on the recommendations of multiple community stakeholders, which included representatives from our CAB. We recruited the participants using REB-approved flyers and recruitment messages posted on the websites, listservs, social media outlets, and physical premises of our numerous community collaborators and supporters, such as not-for-profit LGBTQIA+ agencies and ASOs across the province of Ontario. The individuals who expressed interest in participating in our interviews were screened and included in the larger study if they self-identified as MSM, were 40 years of age or older, and were living in Central or Southwestern Ontario, regardless of their HIV status, as long as they were willing to confidentially disclose their HIV status for the purposes of completing our study’s participant sociodemographics. Our research team and community partners made a valiant effort to recruit a diverse range of participants in terms of their age, race, ethnicity, the geographical location of their residence, how they identified in particular as MSM (e.g., gay, bisexual, pansexual, queer, two-spirited, or simply as MSM), and gender identity. However, despite our best efforts, we were only able to recruit and include in our study three TMSM who met our specific inclusion criteria, all of whom we described and referred to in this article under the pseudonyms Aki, Bailey, and Cameron, and using the pronouns “they”, “them”, and “their”, in accordance to their personal use of pronouns. All three participants have lived in Downtown Toronto for at least five years, and were very familiar with its LGBTQIA+ community and the health and social services it uses, particularly the services that have helped them to meet their own needs.

After receiving comprehensive information about our study, each of the three HIV-negative, middle-aged and older TMSM provided written consent prior to participating in our interviews. They were each interviewed by the first author and one of the two peer researchers in roughly one hour-long sessions. All of the sessions were digitally recorded and held at either the office of one of the interviewers or in a secure meeting room of a community-based organization of the participant’s choosing. The interviews followed a semi-structured interview guide, which was developed and refined by our research team in collaboration with our CAB and community partners. The semi-structured interview guide utilized open-ended questions for the purpose of exploring distinct areas of research interest: (a) the lived experiences of the participants; (b) factors, strategies, or sexual health practices they employed that promoted their resilience to the clinical and social impacts of HIV; and (c) the reasons why they believed these factors, strategies, or practices promoted their resilience to HIV. Participants received compensation in the form of CAD25 cash for their time and participation. The peer researchers transcribed the interviews verbatim, and the first author reviewed each of the transcripts to confirm their accuracy prior to the thematic analysis of the case studies’ data [58,59].

## 3. Case Studies

### 3.1. Aki

Aki is a 48 year old East Asian migrant who moved to Toronto several years prior to participating in our study. As a trans man who engaged in sex work for a few years, Aki’s awareness of information on HIV risks and prevention interventions has been primarily based on the copious materials they studied from sexual health clinics that regularly provided brochures on safer sex practices.

At the very beginning of their interview, Aki reported experiencing persistent financial struggles, which began as early as the first year they migrated to Canada. They moved to Canada to escape discrimination and persecution from their country of origin, and without a lot of transferrable skills, subsequently experienced difficulty obtaining work in Toronto. Aki shared, “I didn’t make a lot of money. Eventually, I ended up working on the streets. I did that for a few years to survive”.

After several years of struggling financially, Aki was able to finally catch breaks with the help of community-based agencies and many referrals to social services. Aki developed a strong and genuine appreciation for the services and programs they were able to access in Toronto, specifically from international non-profit humanitarian organizations, LGBTQIA+ agencies, regional ASOs, large healthcare institutions, and several local multicultural and community health centers. Over the years, Aki then became slowly and heavily engaged with the LGBTQIA+ community of Toronto, where they were able to receive social support not only from the city’s healthcare and social services, but also from queer and trans folks from the LGBTQIA+ community—social support that was essential to building their overall resilience.

When they were asked about their personal sexual experiences and practices during their interview, Aki talked to us about their initial difficulties finding the right spaces in the earlier years of their stay in Toronto, but then recalled how they gradually got into kinky sex, “It was a few years back when I began exploring kinky sex—bondage and discipline, dominance and submission, sadomasochism (BDSM), fetishes, role play, other stuff. Nowadays, it’s the scene I feel most safe in terms of all types of risks”. Aki claimed that it was a relatively easy choice for them to go kinky. For them, engaging in kinky sex would ensure that there was going to be an understanding between them and a prospective sexual partner first before any sexual exploits happened.

Aki then shared with us how they first discovered the role of private house sex parties in popularizing kinky sex, especially in communities they engaged in and enjoyed:

“Before my access to the internet, I already went to different sexual communities. They played a more prominent role back then. Those communities have changed since my access to the internet. Sex parties and gatherings of sexually open people. Way back, there were just free-for-all orgies. Now, because of the internet, all the kinky people, those into BDSM and role playing, they gather and show up every Monday night, and they get to choose and go home with whomever they want after firmly setting expectations and ground rules”.

Before discovering kinky sex at house parties, in the same way as many other MSM, Aki was engaged in spaces such as bathhouses, gay bars, and online apps to meet other men, and potentially initiate sexual encounters. However, they soon learned that they were not very comfortable with the different scenes in such spaces, especially because of the liberal use of alcohol and drugs in certain bars and bathhouses that could potentially increase physical and sexual health risks for them as a trans man. Aki reported:

“I have seen a lot of cis gay men in bathhouses do heavy drugs. In the kinky sex I’ve been part of, there’s maybe some drugs, but certainly not to the extreme extent of how I’ve seen it been used in bathhouses. I can confidently say, it’s not anywhere nearly as prevalent and concerning in the kinky sex parties I’ve joined”.

At the kinky sex parties, Aki has progressively relied on deliberate partner negotiations to keep safe in terms of both their physical wellbeing and sexual health. Related to this, Aki expressed their great appreciation for Downtown Toronto and the MSM who live in it, “I have visited and travelled all over Canada since I moved here, and the most accessible house parties for kinky sex are definitely in Toronto”. They also expressed how vital to their resilience the available healthcare and social services were in Toronto:

“Toronto is the best city! I’m sure it is one of the best cities in the world. In terms of healthcare for queer and trans people, definitely. I learned about things here that I did not even know I needed for my sexual health”.

### 3.2. Bailey

Bailey is a Canadian-born, 40 year old of Western European descent, who strictly speaking, prefers to remain a non-identarian, but would agree to self-identify as part of the MSM community, especially for the purpose of being included in our study. Bailey’s considerable understanding of valuable aspects related to sexual health and HIV are derived from their extensive experiences being in a long-term serodiscordant relationship with an HIV-positive cis gay man.

Our interview with Bailey began with our open-ended question that asked how they were doing in general. Bailey explained how things could be better and shared with us how tight their finances were. Although they expressed that they were aware that many CMSM experienced economic difficulties because of underemployment and unemployment related to stigma and discrimination, they genuinely believed that transphobia, cisgenderism, and prejudice have had a greater negative impact on the job and financial security of many trans individuals in the 21st century. Bailey described the predicament they have been experiencing for years:

“I am marginalized financially. There are not that many opportunities for trans people to make a decent income in large part because of stigma. Cis queer men, most don’t worry about what passes under many employers’ scrutiny. They take one look at me, and no. At this point, it’s minute to minute, day to day. I’m managing poverty by finding research studies that I can participate in that will pay for my groceries. That’s today. Other times in my life, I’ve been able to manage that differently”.

When we asked them what they believed helped them thrive over the years, Bailey’s response was almost immediate. They have long recognized the value of having a robust involvement in the LGBTQIA+ community that provides the support they need:

“I’ve been extremely socially engaged. Learning [through social interaction] has been my biggest way of growing and finding supports. I am a bit of a sex radical and I’ve gone to many sex conferences. Gatherings with many different types of people, many different combinations. I’ve gained a ton of skills that I now recognize are almost completely impossible to replicate organically. These were skills I built to address short, medium, and long term issues [I have had as a trans person]”.

In addition to appreciating the value of social engagement and support from friends to promoting their wellbeing, fortitude, and HIV resilience, another factor that Bailey also appreciated was the support of community-based agencies, organizations, and health and social services. In particular, they highly appreciated what the not-for-profit LGBTQIA+ agencies, ASOs, community health centers, and other Toronto health and social services brought to the table. Bailey discussed their strong connection to community resources:

“One of the main reasons I’m alive is because I’m connected to community resources that will address specific issues that I have. Those are always changing. So, [an LGBTQIA+ non-profit agency] feeds me twice a week. That’s my source of food security. [A community health centre] is also where my family physician is, and my doctor helps me address my sexual health risks. I would not say that GPs have the capacity to deal with the kind of complexity that I’m living, but generally, they are a reliable source of support”.

When our interview moved to the topics of sexual spaces, risks, encounters, and practices, Bailey began to talk about their experiences with going to bars and bathhouses several years back, as well as using apps to meet other MSM. Bailey was quick to point out that their experiences with these spaces were associated with an abundance of caution and quite short-lived because not only were they not as comfortable being in these spaces, but they also recognized that these spaces posed higher physical and sexual health risks for them as a trans man. They recounted some unpleasant experiences:

“I have been on apps, but apps can be dangerous for me as a trans man. I don’t use them the same way that a cis gay man would. I make sure that I’m out on my profile as trans so that no one can ever claim that I did not tell them. That’s one of the ways trans people get killed in sexual encounters. I work my way back from that reality. People still don’t see what they don’t want to see. People will get very angry with me if they later find out that I’m trans. They say things like, “You should have told me sooner”. But I’ve already told them 3000 times. It’s when it registers with them that it can become risky, or even violent”.

Bailey then explained the strategies they employed to mitigate their personal risks:

“On my online profile, I have everything people need to know about why they should contact me if they want to have sex with me, and what kinds of sex I have. I make sure my profile is clear. I do not share face pics with somebody I have not met in person. So many people will only have sex with someone whom they have exchanged pictures with. For safety, I don’t do that. I make it clear that no unsafe sex will happen. But we can have any kind of sex we want as long as it’s safe. I assume everybody is HIV-positive, or I think people may be poz, but do not know it”.

In the course of explaining how they have been able to keep safe during sexual encounters, Bailey started sharing with us their apparent preference for engaging in kinky sex. Since preliminary conversations are part and parcel of identifying and establishing the boundaries of the kinky sex that they were going to engage in with other MSM, Bailey emphasized the value of having a mutually expected and accepted safety step that was built into going kinky. They made the connection between kinky sex and the lowering of HIV risks clear for us in their interview:

“First of all, I have sex with people. But yes, I do have kinky sex with a lot of [cis gay] men. I think HIV is a disease that gives us all an opportunity to think communally. It is not a disease that is specific to only certain individuals, even though it does affect us very individually. [With HIV,] it’s having that understanding that an exchange of bodily fluids can lead to negative impacts on your immune system. So you then realize, in kinky sex, pleasure does not always have to be about penetration”.

Bailey provided more details on what went on during private house sex parties, and why they found good reasons to pursue kinky sex in such parties:

“One of the house sex parties that I go to is all kinky men who have sex with men. It’s every month, and it’s always different people from around the city. That is one of the places where I develop skills about that particular kind of sex. It’s all very sexually rich. I see at least ten different men having sex once a month. One of my safety fallbacks in these house sex parties is that I’m having anonymous sex with people …there’s the unsafe side vs. the safe side. The unsafe side is that if some folks find out I’m trans, they can get very upset. The safe side is, if they don’t want to have sex with me, there’s a room full of other people they can have sex with and move on. Same goes for me. The stakes are also actually lower [in terms of HIV risks], even if there’s a rejection factor involved”.

According to Bailey, the more conversations prior to engaging in kinky sex were had, the safer it was for them. There is an openness to the conversations that eventually lead up to deliberate negotiations between prospective sexual partners, which may or may not work out. If the deliberate negotiations succeed, consent follows between partners, and initial trust is established. Bailey also narrated their kinky sex experiences related to deliberate negotiations:

“All my safer sex practices come from my kinky sex practices, which are all about consent and negotiations. Communication and heightening pleasure. I’m maintaining the wellbeing of all people involved. Being clear about which things can be protected and nurtured, and which things can’t. My safer sex practices are due to philosophies and values [of kinky sex]. I’m never in a situation where I accidently don’t have safer sex. For many of us [trans men], being kinky has been an important component of our survival”.

There were other facets of kinky sex that were vital to the promotion of their HIV resilience. Based on their personal experiences, they were able to encounter more gender-affirming CMSM during their kinky sex exploits. According to Bailey:

“I’ve had a lot of socialization experiences during my sexual encounters. A lot of the [positive] acknowledgements of my gender in the wider social and sexual sphere have been really affirming, especially since I transitioned. Almost every [kinky sex] sexual interaction since I transitioned has been about gender-affirming relations”.

During their sexual encounters in the earlier years at spaces such as bathhouses, bars, and online apps, where deliberate negotiations between prospective sexual partners were not as valued, prioritized, or even considered, Bailey noted that there were less opportunities to meet CMSM who made them feel accepted or gave them a sense of belonging in the MSM community. They shared that they were much more likely to experience a greater sense of belonging and/or an affirmation of their gender in the spaces where kinky sex was the norm.

Another vital facet of engaging in kinky sex that Bailey emphasized is their greater appreciation for the discipline to avoid the use of alcohol and drugs during deliberate negotiations and kinky sex in order to mitigate sexual health and HIV risks. Bailey explained this decision to avoid alcohol and drugs while initiating and then engaging in kinky sex:

“I guess one of the things I would have to say is that it goes back to the communication and clarity. I have not been a person who was having sex while high. I’ve used recreational drugs. But I don’t have sex while doing it. That goes back to the kinky sex. Safe, sane, and consensual. You can’t give consent if you are drunk or on drugs”.

Beyond having provided the opportunity to meet, socially engage, and have sex with more trans-affirming CMSM, Bailey reported in their interview that Downtown Toronto was a prime location like no other place in Canada. They reported that in terms of accessing trans-competent health and social services, other cities could not match what Downtown Toronto offered, “I use every resource accessible to me. That’s why I live in this postal code. It’s the most resource rich area in the country. Outside of the Downtown core, most everything I need would be difficult to access”. 

Despite their laudable access to many health and social services, Bailey was keen to draw attention to the fact that, based on the HIV services and programs they have encountered and had the opportunity to evaluate with their long-term partner, most prevention interventions in the Ontario HIV sector were centered on the contexts, risks, practices, and needs of CMSM. Bailey expressed their frustration as they explained:

“Unfortunately, in most community health centres and clinics, their attention is very much focused on cis gay men and their sexual health. So that’s where all the efforts for STI and HIV prevention programming go. The type of sex I have, it doesn’t always line up with traditional notions of condom use and the transmission of HIV. That has been a problem. I learned a lot of facts that I didn’t know previously as I engaged more and more in kinky sex. When I sought out information about how we could protect ourselves, I found that all agencies just had a script. Here’s how gay men have sex. Here’s how we convey condom use as safer sex. Everything else, just goes away. That re-traumatizes trans men who have sex that does not line up exactly with sex of most cis gay men”.

In addition to a lack of consideration for the specific contexts, risks, practices, and needs of TMSM, Bailey added that many healthcare practitioners and service providers made assumptions that could prove harmful to them as a trans man:

“With me being trans, the assumption of many in healthcare was that my sexual partner and I both have penises that ejaculate, and all that top bottom shit that didn’t apply to many of us at all. That’s why PreP is bothering me. It’s the new condom campaign. It still fucks up everything in my life…it’s traumatic for me to constantly hear the terms ‘gay, bisexual, and MSM’ in healthcare…with my life constantly having to be subsumed under that MSM category, and yet, as a trans man [who has sex with men], still be largely excluded. People in healthcare need to understand that what they routinely offer in their HIV prevention programs for cis gay men don’t always apply to trans men”.

Bailey described that, in addition to the usual emphasis on the use of condoms and PrEP, there is a need to increase the focus on knowledge and skills for promoting deliberate partner negotiations and the benefits of engaging in kinky sex in HIV prevention interventions tailored for TMSM. According to Bailey:

“As an example, I have yet to see much more healthcare or health services sites on the internet primarily focused on communication and negotiation skills, particularly in the context of trans men’s sexual health and kinky sex. With regards to safer sex for trans men who have sexual experiences with other men, nobody is really talking about it”.

### 3.3. Cameron 

Cameron is a 53 year old French Canadian whose knowledge about sexual health practices and HIV risks and treatments comes mostly from several years of volunteering in Toronto LGBTQIA+ agencies and ASOs, and having very close friends who are MSM living with HIV.

Cameron began their interview by sharing with us how long they have been actively part of the LGBTQIA+ community of Toronto and their heartfelt gratitude for having several long-time friends from the community who have provided them with immense emotional and practical support over the years. According to Cameron:

“In terms of friends, I have had a ton of support. I was always very engaged, and I was also privileged in the sense that I was able to navigate the [Ontario] healthcare system with the help of friends from the community. I knew [other trans] people who knew people so I could get good counselors, good doctors, and care and services I needed”.

It has been this kind of consistent social engagement that has connected Cameron to bankable support systems within their chosen community. With respect to promoting their HIV resilience, Cameron expressed that community resources were vital to overcoming their specific challenges as a trans man and helping them thrive. Having spent so many years living in Toronto, Cameron has seen the city go through many changes, particularly in the Church and Wellesley area (i.e., “the village”), and how these changes have affected their community since the HIV epidemic began in the early 1980s. They explained how these changes have also personally affected them and the decisions they have made along the way. Cameron shared their experiences and the reasons why they have made certain calculated choices:

“Back then, I did go to bathhouses, but I didn’t really feel comfortable there. Being a trans man, I didn’t get bottom surgery. So I was kind of uncomfortable about going to bathhouses. Nowadays, usually, a bar I go to is the Black Eagle. There’s a monthly event called ‘ruff house’, not sure if you’ve heard about it. It’s like a BDSM sex space at [a club that is no longer operational]. So it happens once a month, and that’s a place where I can go where I can actually come out as trans, and it does not seem to be a problem in the kinky BDSM community. It seems to be less of a problem amongst cis men in that space, and I think, people there are a bit more open. I’ve also been online, like most everyone else. I’ve had some bad experiences of feeling objectified. Like, some people who don’t even know me asked me really intrusive questions about my genitals. So you know, I just kind of rather meet in person and talk first”.

Then, Cameron shared how later in life they eventually found private house sex parties, which have become crucial to keeping kinky sex and deliberate partner negotiations as enduring, viable, safer options, specifically for a trans man such as them:

“I’ve tried very hard to avoid occasions where there will be spur-of-the-moment sex. That’s why I prefer going to private [house] sex parties, where the circumstances are more controlled and contained…where there is an understanding among everyone in attendance. In terms of being with someone in private, it’s where I can develop more communication and connections, and really, the conversations don’t have to take that long!”

The deliberate partner negotiations may involve numerous aspects such as preferences, roles, and safety strategies, but almost always involve addressing sexual health, STI, and HIV risks, even if the negotiations are short and quick. Cameron described their experiences related to building trust through deliberate negotiations prior to kinky sex at private house sex parties:

“In these private sex parties, there is more talk and developing trust. There’s also the conscious elements of the power dynamic and power cycle, which makes it more exciting! Kinky sex is like a different flavor of sex, where we can embed more safeguards for trans folks like me”.

When their sexual encounters were mutually satisfying, the experiences were not only safe, but they also became truly gender-affirming for Cameron. They reported, “I haven’t had sex with trans men. I’m mostly attracted to cis men. When I suspect that they want to have sex with me, it seems like they think I’m hot. This is when I feel most accepted”. 

It was this kind of confidence and positivity that they developed when they experienced gender-affirmation from prospective kinky sex intimate partners at house parties, in addition to the increased feelings of safety and control they gained in the process. Although there were MSM who still drank alcohol and took drugs in private house sex parties, Cameron and the TMSMs who they knew who engaged in kinky sex intentionally remained alcohol- and drug-free in order to feel safe, stay in control and sober during deliberate negotiations, and clear headed to provide consent and build trust. Cameron shared how they felt they already had enough safety issues to contend with in the first place, and why it would be unwise for them to use alcohol or drugs that would decrease their ability to efficiently navigate deliberate negotiations prior to kinky sex, as well as provide or gain consent, “I’ve definitely seen it, especially in bathhouses. I don’t participate in it. I’m not interested in drugs. I drink socially…that’s about it. I already have enough safety concerns so I don’t want to compound them with drug use during sex”. 

As we continued to discuss sexual exploits and access to safer options, Cameron, who was originally from the province of Quebec, explained why they were first attracted to the promise of living in Downtown Toronto, “Ultimately, I found [a city in the province of Quebec] was too small a community for me to be comfortable in, and not as accepting of trans folks like me. I wanted to meet guys, and overall, it had less open-minded men than Toronto”. They shared details on how they eventually recognized just how ahead of the pack Toronto was in terms of accessible health and social services for trans men, and how important this was to them:

“When I was in Quebec, it took me a really long time to find the right doctor. It was really hard to find any family physician who would take me in as a patient. Here in Toronto, it was easier to find a doctor, there’s just so many…trans-friendly doctors too. Seriously, I found a family physician within two weeks of moving here. But then, he later moved out of town. It didn’t matter since I immediately found another doctor from the same clinic [at the gay village]. There are more health and support services for trans people here as well. Hands down, for trans people, Toronto is the place to be”.

However, Cameron also described how disappointed they were to find out that most of the current HIV prevention interventions and campaigns they encountered were predominantly focused on the use of condoms and PrEP, which they acknowledged were very important, but noted to be geared more specifically to address concerns more pertinent to CMSM. Although they recognized that the vast majority of MSM in the province were cis, they also wanted to emphasize that prevention interventions focused on condoms and PrEP, that were targeted efforts to promote HIV resilience among CMSM, were not always as pertinent to them as TMSM. Cameron felt that if more MSM, especially CMSM, were made aware and informed of other sexual health practices, particularly those that line up with the practices of TMSM, then this would promote the resilience of TMSM to HIV. Additionally, they suggested that it would be essential for healthcare and service providers to learn more about the actual STI and HIV risks of TMSM. Cameron believed that many providers still thought that all trans men were at low risk for HIV and other STIs. Cameron reported:

“Even though I was kept being told by people in health services that my risk for HIV was very low, I still tested for it every six months. I practiced safer sex with cis gay men, but I just felt better getting tested. So I got tested a lot!”

According to Cameron, what is harmful to TMSM is that many of the HIV services and programs, on one hand, treat TMSM as individuals with low sexual health risks that warrant little public health concern, and on the other hand, lump them together and combine them in the same category as CMSM, as if TMSM had no distinct sexual health practices and needs. They shared from their own experiences that even well-meaning healthcare and service providers did not have as much awareness that TMSM need to have HIV prevention interventions that are customized to their own sexual health risks and practices. Although the efforts to focus on promoting deliberate partner negotiations have over the years seemingly been almost sidelined in HIV services and programs in favor of a focus on promoting the use of condoms and/or PrEP with the HIV risks and sexual health practices of CMSM in mind, Cameron clearly believed that there is a need to seriously rethink and reconsider what will work best for HIV prevention interventions specifically customized for TMSM. 

## 4. Discussion

In addition to the stereotypes, prejudice, stigma, and discrimination they have regularly faced from society as part of an umbrella HIV risk-category population, TMSM and CMSM have also historically shared other experiences and contexts worth noting. For instance, as we learned from the case studies, particularly from the experiences shared by Aki and Bailey, TMSM may also persistently face other HIV acquisition risk factors that commonly affect CMSM, such as financial struggles due to underemployment or unemployment. Several studies have documented the economic hardships experienced by many MSM (including TMSM), which inadvertently lead some MSM to engage in transactional sex work for survival [8,60,61,62,63,64]. Related to their capacity to promote their resilience to HIV, engaging in sex work has been found to increase the sexual health and HIV risks of MSM [15,16,35]. Other critical factors that significantly impact the capacity of both TMSM and CMSM to promote their HIV resilience are their perceived social support and accessible community resources that help them meet their most basic needs. In the case of TMSM, this reliance on and great appreciation of social engagement, social support, and accessible community resources were distinctly exemplified by the experiences and sentiments described in all three case studies. Several research studies have emphasized the benefits gained by MSM and trans individuals from having perceived social support from LGBTQIA+ friends [65,66,67,68,69], and strong connections with gender-affirming communities and sexual health care [46,47,51,52,53,65], especially those that prioritize the elimination of cisgenderism and transphobia [70]. They have also emphasized the need for such HIV services and programs to create sustainable opportunities to increase the financial security of TMSM, as well as develop more opportunities for TMSM to receive gender affirmation and social support from accessible community resources. 

However, aside from the factors that TMSM have in common with CMSM that impact their capacity to promote their resilience to HIV, our three case studies illustrated that TMSM also have experiences, strategies, and sexual health practices that they favor or choose to exercise in order to lower their sexual health and HIV risks, which not as many CMSM seem to have or carry out as often (please see Table 1 for the thematic commonalities found among the three TMSM case study interviews). According to our participants, whereas many more CMSM reportedly frequent bathhouses and bars, as well as rely on online dating or hook-up apps to meet other MSM, as TMSM, they have personally had a stronger inclination to invest their efforts into seeking spaces that allow them to engage in kinky sex. This is because with kinky sex, there is the built-in protective strategy of going through deliberate negotiations between prospective sexual partners prior to engaging in sexual activity.

As a crucial point of clarification, when kinky sex was mentioned or discussed by the three TMSM participants in our case studies, they were referring to the catch-all term for a range of sexual practices that included BDSM, role play, fetishes, and other sexual practices that essentially involved consensual deliberate partner negotiations, as well as pleasurable experiences for all of the parties involved. Although it is beyond the scope of this article to present an elaborate discourse on kinky sex that explicitly discusses the complex relationship between kink and trans masculinity, it is important to acknowledge that there is published academic literature available that discusses this relationship in greater detail [71,72,73].

Based on the experiences of our three TMSM participants that were narrated in our case studies, deliberate partner negotiations prior to kinky sex foster clearer communication, establish explicit consent, and build trust, which are all important tactics that could be employed, in addition to the other risk-mitigating strategies that have been more traditionally perpetuated by CMSM-focused HIV services and programs such as the promotion of HIV status disclosure, condom use, and PrEP use [1,8,9,62]. In the last two decades, deliberate negotiations, clearer communication, explicit consent, and the forging of trust have gradually been entrenched and deeply embedded as part of evolving frameworks (i.e., safe, sane, and consensual (SSC); risk aware consensual kink (RACK); and caring, communication, consent, and caution (4cs)) that have informed and helped establish the culture and norms of BDSM, role playing, and other forms of kinky sex [74,75,76].

The practice of having deliberate negotiations between prospective or regular sexual partners prior to engaging in sexual activity to mitigate sexual health risks, specifically the risks of HIV transmission and acquisition, has been thoroughly researched, and even supported by HIV prevention intervention services and programs, for over four decades [77,78,79,80]. In particular, studies have examined the strategies, tactics, and challenges associated with using timely communication to obtain consent, often to engage in condomless anal intercourse, among MSM [77,78,79,80]. However, more recent research has shown that since the advent of PrEP, previous safer sex norms among MSM have arguably been disrupted as the centrality of condom use in HIV risk reduction has apparently decreased and new patterns of deliberate partner negotiations have consequently been introduced [81], likely including new patterns of deliberate partner negotiations involved prior to engaging in kinky sex. 

Two facets of engaging in deliberate partner negotiations and kinky sex that our participants emphasized as crucial to promoting their HIV resilience were the significance of experiencing the validation of their gender identity from trans-affirming sexual partners and remaining sober during negotiations and sexual activities to ensure clear communication and explicit consent. Bailey and Cameron, in particular, detailed specific experiences and perspectives that underscored their personal appreciation for gender-affirming sexual encounters as well as their strong preferences to remain sober and clear headed during these sexual encounters. These two facets have already been previously explored and documented as research has already covered the benefits of receiving social gender affirmation during sexual interactions [45], and the complications brought about by alcohol and/or drug use during deliberate negotiations and sexual exploits that follow [77].

Among the many important messages that our participants conveyed in our case studies, a very salient point that they made concerned the need for having more accessible trans-focused and trans-competent care and services to promote their resilience to HIV. Despite their appreciation for being able to live and access many of their much-needed resources in Downtown Toronto, Bailey and Cameron discernibly expressed concern for the predominant focus of HIV prevention intervention services and programs they could access on the specific contexts of CMSM and the pressing need for more trans-focused and trans-competent health and social services. It has already been established that TMSM are at elevated risk of HIV, and therefore, they require access to HIV services and programs that are both tailored specifically for them [82], and preferably amenable to further adaptation [83]. In fact, TMSM have been found to have unique challenges related to accessing health services and the mitigation of HIV risks, ranging from obtaining optimal gender-affirmative care to negotiating safer sex with CMSM [8]. 

In terms of practical supports, free or low-cost HIV testing, condoms, and lubricants, as well as PrEP, are foundational HIV prevention strategies that are geared towards the needs of MSM, yet are often inaccessible to TMSM. In the global context of stigma and poor healthcare access, TMSM face additional barriers to HIV prevention services since many healthcare and social service providers are unaware of, unfamiliar with, or insensitive and unresponsive to their risks and needs [2,62]. Research has shown that TMSM have reported inadequate access to basic prevention services and that they have been less likely than CMSM to have access to HIV testing, condoms, lubricants, and PrEP [2,62]. This indicates the need to enhance access to basic HIV prevention services for TMSM, including MSM-specific services [2]. The barriers to such access have included both general and trans-specific difficulties in accessing sexual health services, a lack of trans health knowledge among testing providers, limited clinical capacity to meet HIV testing needs, and a perceived gap between trans-inclusive policies and their implementation in practice [1]. Many TMSM still lack adequate information about PrEP and have encountered significant barriers to accessing PrEP. They have also reported that many providers avoid important discussions regarding sexuality and contraception related to the contexts of TMSM [62].

Beyond drawing attention to the issue that TMSM have perceived additional barriers to gaining access to HIV prevention intervention services and programs compared to their cis counterparts, the lived experiences of our participants described in our case studies support the data from prior research that has highlighted the need to develop and provide HIV services and programs that would meet the unique needs of TMSM. Therefore, more detailed, specifically designed studies, services, and programs for TMSM are necessary to complete what we do not know about their sexual health needs, HIV risks, challenges, and strengths [8,24,83].

In the adaptation, tailoring, and creation of HIV prevention intervention services and programs for TMSM, the changes and improvements would first need to be explicitly designed to help prevent significant issues such as the exclusion of trans perspectives and preferences, as well as the promotion of gender dysphoria among TMSM, at the most fundamental levels. These changes and improvements would need to ensure the implementation of supportive language and pronoun use directed by the trans individuals; use of trans individuals’ preferred body terminologies or general terms that do not gender the body in services and program pamphlets, resources, and discussions; promotion of supportive interpersonal engagements of service providers; and hiring more trans individuals on staff [84]. Integrating sexual health information “by and for” TMSM into health services, such as some of the information shared by our participants in the case studies we presented in this article; involving peer support from a more trans-affirming community; addressing the psychological wellbeing of TMSM; and increasing internet-delivered information for TMSM and their sexual partners, have been seen by TMSM as important aspects for improvement and innovation in future HIV prevention intervention design and delivery [25].

Trans men often encounter resistance and reluctance pertaining to their healthcare needs, and are routinely left out of representation not only in healthcare and research, but also in education [85]. Several researchers have indicated the dire need to raise awareness among, and provide appropriate education and training on the contexts, sexual health and HIV risks, and most useful and effective safer sex practices and prevention tactics (e.g., communication and negotiation skills) specific to TMSM, to not only the healthcare and service providers in the HIV sector, but also the CMSM, TMSM, and other trans individuals [14,15,25,84,85,86,87,88]. Some researchers have pointed to the lack of relevant HIV risk education and re-education in TMSM communities, especially around the lack of condom use and other practical evidence-based preventive strategies [14,15]; while other researchers have gone as far as advocating for promoting these awareness and training efforts to be incorporated into the post-secondary education curricula of future health professionals in order to ensure future gender sensitive and affirmative care in sexual health [87,88]. Collectively, these recommendations could potentially help rectify the lack of awareness and knowledge on, and neglect of the prevention contexts and preferences of TMSM in, HIV services and programs, which our participants called attention to in our case studies. These recommendations have also brought to light what we believe is the shared responsibility of TMSM, CMSM, their healthcare and service providers, and larger communities; the shared responsibility to redress the inattention to the HIV prevention needs of TMSM so that TMSM are not left out in the ongoing mission to end the HIV epidemic.

Ultimately, the HIV prevention intervention services must tailor their programs and efforts to focus on and address concerns more pertinent to TMSM and their MSM partners [8,16,24,83], such as promoting and generating more spaces that: (a) help improve the financial stability of TMSM; (b) allow for and facilitate deliberate partner negotiations and kinky sex; (c) promulgate clearer communication and the establishment of explicit consent; (d) endorse and encourage gender affirmation and validation; and (e) educate other MSM, providers, and the larger communities about the kinds of sex that TMSM engage in and the HIV prevention strategies, services, and programs that will be the most suitable and effective for them. It would also be critical for HIV services and programs to recognize that not only do they need to disaggregate trans women from MSM in the development and creation of HIV prevention strategies to mitigate HIV risks, as some researchers have suggested [31], but based on the perspectives and lived experiences of our participants that we described in our case studies, they also need to disaggregate TMSM from CMSM in the design and implementation of their HIV prevention efforts and care programs.

## 5. Conclusions

We have learned that as much as TMSM could greatly benefit from the MSM-focused HIV prevention interventions, they still require services and programs that promote spaces and sexual health practices that they believe would make them feel more validated and affirmed, healthier, and safer. Incorporating information in HIV services and programs that places greater emphasis on deliberate partner negotiations, such as those that are highly valued in the practice of kinky sex, is only one step towards explicitly supporting TMSM. They also need other MSM, their healthcare and service providers, and the larger LGBTQIA+ communities to become more aware, acquire more tailored education and training, and learn and care more about their preferences, needs, challenges, and strengths as TMSM, so that everyone could continue to help promote their resilience to HIV in solidarity.

In order to promote the spaces and sexual health practices that would help TMSM feel more validated and affirmed, healthier, and safer, future research projects could potentially conduct studies that would not only further investigate more of the resilience-building factors that could be incorporated into HIV services and programs specifically dedicated to the contexts and needs of TMSM, but also actively and collaboratively involve TMSM in their research efforts. In the future, the scholars working on this research agenda would need to prospectively identify and implement innovative ways to meaningfully involve TMSM in their studies, perhaps by considering community-based participatory research as well as strengths-based, capacity-building approaches that would include TMSM from the community as the key opinion leaders, community advisory board members, knowledge and cultural brokers, and/or peer researchers who are significantly invested in the work of forward-thinking research teams. These approaches could conceivably help obtain even more useful knowledge that was not garnered by our study, primarily due to the lack of involvement and direct input of TMSM in its research process and conduct.

## Figures and Tables

**Table 1:** Thematic commonalities found among the TMSM case study interviews.

Commonalities found among all Three TMSM Case Study Interviews:
Participants’ appreciation for social engagement and support, and accessible community resources Deliberate partner negotiations seen as useful for fostering risk-mitigating strategies
Commonality found in the case study interviews with Aki and with Bailey:
Participants’ experiences with financial struggles due to underemployment and unemployment
Commonalities found in the case study interviews with Bailey and with Cameron:
Participants’ appreciation for gender-affirming sexual encounters Participants’ strong preference to avoid alcohol to remain sober during sexual encounters Participants’ strong preference to avoid drugs to remain clear-headed during sexual encounters Participants’ concern for the predominant focus of HIV prevention intervention services and programs on the specific contexts of CMSM and the pressing need for more trans-focused and trans-competent health and social services
